# Comparative optimism about infection and recovery from COVID‐19; Implications for adherence with lockdown advice

**DOI:** 10.1111/hex.13134

**Published:** 2020-09-27

**Authors:** Koula Asimakopoulou, Vera Hoorens, Ewen Speed, Neil S. Coulson, Dominika Antoniszczak, Fran Collyer, Eliane Deschrijver, Leslie Dubbin, Denise Faulks, Rowena Forsyth, Vicky Goltsi, Ivan Harsløf, Kristian Larsen, Irene Manaras, Dorota Olczak‐Kowalczyk, Karen Willis, Tatiana Xenou, Sasha Scambler

**Affiliations:** ^1^ Centre for Host Microbiome Interactions, Faculty of Dentistry Oral & Craniofacial Sciences, King's College London London UK; ^2^ Centre for Social and Cultural Psychology, KU Leuven Leuven Belgium; ^3^ School of Health & Social Care University of Essex Colchester UK; ^4^ University of Nottingham Nottingham UK; ^5^ Medical University of Warsaw Warszawa Poland; ^6^ The University of Sydney Sydney NSW Australia; ^7^ Ghent University Gent Belgium; ^8^ University of New South Wales (UNSW) Sydney NSW Australia; ^9^ University of California, San Francisco , Department of Social and Behavioral Sciences La Jolla CA USA; ^10^ Universite Clermont Auvergne Clermont‐Ferrand France; ^11^ CHU Clermont‐Ferrand, Service Odontologie Clermont‐Ferrand France; ^12^ Metropolitan College Athens Greece; ^13^ Oslo Metropolitan University Oslo Norway; ^14^ University of Copenhagen Kobenhavn Denmark; ^15^ La Trobe University Melbourne Vic. Australia

**Keywords:** comparative optimism, COVID‐19, lockdown, risk perceptions, unrealistic optimism

## Abstract

**Background:**

Comparative optimism, the belief that negative events are more likely to happen to others rather than to oneself, is well established in health risk research. It is unknown, however, whether comparative optimism also permeates people’s health expectations and potentially behaviour during the COVID‐19 pandemic.

**Objectives:**

Data were collected through an international survey (N = 6485) exploring people’s thoughts and psychosocial behaviours relating to COVID‐19. This paper reports UK data on comparative optimism. In particular, we examine the belief that negative events surrounding risk and recovery from COVID‐19 are perceived as more likely to happen to others rather than to oneself.

**Methods:**

Using online snowball sampling through social media, anonymous UK survey data were collected from N = 645 adults during weeks 5‐8 of the UK COVID‐19 lockdown. The sample was normally distributed in terms of age and reflected the UK ethnic and disability profile.

**Findings:**

Respondents demonstrated comparative optimism where they believed that as compared to others of the same age and gender, they were unlikely to experience a range of controllable (eg accidentally infect/ be infected) and uncontrollable (eg need hospitalization/ intensive care treatment if infected) COVID‐19‐related risks in the short term (*P* < .001). They were comparatively *pessimistic* (ie thinking they were *more* at risk than others for developing COVID‐19‐related infection or symptoms) when thinking about the next year.

**Discussion:**

This is one of the first ever studies to report compelling comparative biases in UK adults’ thinking about COVID‐19.

## 1 BACKGROUND

Until a vaccine and/or an effective cure for COVID‐19 becomes available, battling the current pandemic will critically depend on how well people follow behavioural advice to adhere to lockdown restrictions, adhere to social distancing rules and engage in effective personal hygiene. It has been reliably established that peoples’ perceptions about a situation influence their behaviour.^1^ Understanding people’s thinking about COVID‐19 risk in this pandemic is thus critical in understanding and predicting COVID‐19‐related behaviour in future.

One well‐established phenomenon in risk perception is comparative optimism. Comparative optimism is the belief that negative events are less likely to happen to oneself than to others.^2^, ^3^ The majority of people of all genders and ages show comparative optimism for a wide variety of risks, including many health hazards.^4^, ^5^ For example, people believe that they are less likely than others to be involved in a car accident, to experience a divorce, to fall victim to a crime or to lose their job.^2^, ^3^ Although comparative optimism is impressively robust, some systematic variation has been observed. One well‐documented finding is that comparative optimism is more pronounced for risks that people deem controllable, such as lifestyle‐related health problems.^6^, ^7^ In this paper, we explore the occurrence of comparative optimism in relation to COVID‐19‐related behavioural risks.

Controllability of COVID‐19 risk has been an important factor of the UK Government Public Health advice. At the start of the UK lockdown, the Government communication focused on the idea that staying at home would have direct positive impacts on curbing COVID‐19 transmission. The slogans ‘Stay at Home, Protect the NHS, Save Lives’, recently replaced by ‘Stay Alert, Control the Virus, Save Lives’, had at their heart the idea that this pandemic was controllable by individuals taking personal action.

At the same time, research has shown that greater perceived controllability of an event enhances the likelihood of greater comparative optimism,^6^, ^7^ and so we suggest it is likely that recommendations encouraging protective behaviours may be positively associated with enhanced comparative optimism during the lockdown period. If this is the case, we would expect to see high rates of comparative optimism concerning aspects of COVID‐19 that people may judge as being personally controllable.

In sharp contrast, there are aspects of the pandemic that people would arguably perceive as uncontrollable. Whilst in early communications about people succumbing to the virus the UK public were told that people dying tended to have underlying health conditions, later on, it became apparent that the virus was more indiscriminate, also killing people with no underlying conditions.^8^ It is, therefore, likely that people may perceive recovery or not from COVID‐19 as being outside their direct control. If this were the case, we would expect people to show less comparative optimism about the risk of suffering serious consequences once infected, than about the risk of getting infected in the first place.

The question whether people show comparative optimism concerning COVID‐19 is important because of its potential psychological and behavioural consequences. Comparative optimism may have elicited the anecdotally observed lack of compliance with lockdown guidelines in the UK.^9^ Despite overwhelming public support for continued lockdown prior to a safe, gradual loosening of restrictions,^10^ 25% of inhabitants of some areas admitted breaking lockdown rules.^11^ Such failure to comply with lockdown guidelines may have many causes other than comparative optimism. These include, but are not limited to, misunderstanding of lockdown principles, boredom, loneliness, mistrust in policymakers, belief in herd immunity, and/or the desire to alleviate the assumed loneliness or boredom of others. Surveys carried out by King’s College London and Ipsos Mori suggested that by week 5 of the UK lockdown (w/c 20th April 2020), 2 in 5 younger people (18‐25 years) were finding lockdown restrictions extremely difficult to cope with, or expected to find it so in the next 4 weeks.^12^ This is against a backdrop of misunderstanding of guidelines, such as about how often people are allowed to leave the house and what they are allowed to leave the house for.^12^ Alongside these factors, it is likely that people who believe COVID‐19 is less likely to happen to them than to others may infer that their actual risk is much smaller than that communicated in the media, and thus that strict adherence to lockdown restrictions is unnecessary in their case.

Previous research has shown that comparative optimism contributes to risk‐taking.^13^ Greater comparative optimism is known to be associated with more risk‐increasing behaviours.^14^ Conversely, comparative optimism may also positively contribute to people’s mental health^15^ whereby in reducing anxiety, it might enable the fostering of positive relationships.^16^, ^17^ Comparative optimism around COVID‐19 may thus have both desirable and undesirable consequences for the behavioural response to the pandemic.

There is ample reason, therefore, to examine the extent to which comparative optimism occurs in risk perceptions concerning COVID‐19. This paper reports UK data from an online international survey testing the following hypotheses:

1. People are comparatively optimistic about COVID‐19; people will report being *less* likely than others to experience negative COVID‐19‐related outcomes (eg getting infected in the first place) and more likely to experience a positive outcome (ie full recovery if infected).
2. Stronger comparative optimism will occur for those aspects of COVID‐19 that people have been encouraged to view as personally controllable (ie the likelihood of getting infected or infecting others) than for those aspects that they view as being less personally controllable (ie how ill one gets once infected).

## 2 METHODS

### 2.1 Design

We conducted an online cross‐sectional international survey across 10 countries at a time, whilst many countries were controlling people’s movement through various COVID‐19 lockdown restrictions. On 24th May 2020, there were N = 15 084 recorded responses, from people residing in a variety of countries in Europe (eg UK, Denmark, Norway, Sweden, Greece, France, Poland, Belgium, The Netherlands), the United States and Australia. This paper presents data from UK‐based participants only. We report here only on the UK data because the particular way in which lockdown was eased in the UK, focusing on personal responsibility, makes comparative risk perceptions particularly pertinent to the UK context.

### 2.2 Materials

The survey was developed by an international team of psychologists and sociologists. The survey asked questions about comparative optimism beliefs regarding COVID‐19 and about a range of impacts on daily life arising from COVID‐19 Government‐imposed restrictions. For example, participants reported their experience of COVID‐19 symptoms and answered questions about access to resources such as services, people and outside space. They also answered questions tapping into their perception of who had been responsible for transmitting the disease or curbing its transmission. Another part of the survey elicited their perceptions of the extent to which the virus had also provided positive experiences (benefit‐finding), such as appreciating one's family more, or developing the ability to focus on things that matter. Finally, participants reported on unhealthy coping behaviours such as smoking and drinking alcohol. This paper reports on the section of the survey (N = 10 items) assessing comparative optimism about infection by and recovery from COVID‐19.

The comparative optimism questions asked participants to consider ‘the average person of your age and gender’ and to rate how likely it was that a series of COVID‐19‐related events would happen to themselves as compared to that average person. Some events could arguably be considered as being within the person's control (eg How likely is that over the past month you have accidentally infected others with COVID‐19/ that you will get infected with COVID‐19), whilst some were likely to be seen as uncontrollable (eg How likely is that if infected you will need hospitalization/ that you will find yourself in an Intensive Care Unit). Participants answered on 5‐point Likert scales ranging from ‘Extremely likely’ (1) to ‘Extremely unlikely’ (5). Finally, participants reported on a variety of demographic information, including the existence of underlying health conditions identified by the NHS as making them more vulnerable to COVID‐19.

### 2.3 Participants

This paper reports data from a UK sample of adults (N = 645) who completed the survey during the period 24 April‐10 May 2020 8.00 GMT (ie weeks 5‐8 of the COVID‐19 lockdown).

### 2.4 Statistical methods and Power

Cronbach’s alpha analysis was used to assess the scale for internal consistency. Exploratory principal component analysis (PCA) was used to examine whether the comparative optimism scale consisted of identifiable subscales. For this, we used a Varimax rotation, thus not allowing the factors to correlate, and an Eigenvalue of 1. No further analyses relating to the PCA (eg Goodness of Fit) were performed.

Using guidance for analysing comparative optimism data^18^ we converted participants’ responses into a comparative optimism continuous scale from −2 to +2, such that positive scores indicated comparative optimism, zero indicated a perceived likelihood equal to that of another average person of one's age and gender, and negative scores indicated comparative pessimism. We tested the occurrence of comparative optimism/ pessimism on each subscale and then on each individual item through single‐sample *t* tests against a hypothesized population mean of 0.

A repeated measures ANOVA was used to test for differences in comparative optimism across the extracted PCA factors. Gender differences in responses to the three subscales were explored through independent samples *t* tests. All analyses were repeated with and without participants who had self‐identified as being at higher risk for COVID‐19 to ensure that their responses did not bias our findings.

Finally, statistical power was assessed post hoc. The required sample size for a single‐sample *t* test to detect a small effect (d = 0.25) was N = 210. The survey was thus sufficiently powered to detect a small effect, using a single‐sample *t* test, with a hypothesized population mean of 0 and a 95% confidence level.

### 2.5 Procedure

As the data were collected during lockdown, the need to engage in novel yet reliable methods of recruitment defined this process. Potential respondents were invited to participate through researcher networks on various social media including Facebook, Twitter and WhatsApp groups and through direct e‐mail. Contacts within these networks were asked to pass the link to the survey onto their networks, in a snowballing recruitment method deemed appropriate for online research especially with people who would normally experience barriers to participating in research.^19^ When potential participants clicked on the survey link, a Participant Information Sheet (PIS) appeared. This PIS gave information about the topic of the survey, assured participants of anonymity and confidentiality and referred them to sources of mental health support, if necessary. Participants were told they could skip survey questions and exit the survey before completing it in full.

Only participants who actively consented to take part were able to proceed onto the survey. The survey began with the COVID‐19 symptom experience questions, followed by the comparative optimism scale. Demographic questions and those about risk factors were answered last. Participants who reached the end of the survey were thanked, debriefed and reminded of sources of mental health support.

## 3 RESULTS

### 3.1 Scale reliability

The Cronbach's alpha analysis of our data showed that the scale was reliable overall (α = 0.705).

### 3.2 Demographic characteristics analysis

The demographic characteristics of the sample appear in Table 1. In line with data on health information preferences^20^ the sample was predominantly female and White. Although we sought data on years of post‐primary school formal education, 70% of the sample did not respond with the vast majority reporting in ways to suggest they had found the wording confusing. As a result we have not reported this data, although for those who responded numerically, responses ranged from 0 to 23 years. Most of the sample self‐reported being employed. Age was normally distributed with the majority of respondents being aged between 35 and 54 years. The sample reflected the UK population in terms of disability patterns and existence of conditions that would place people at a higher risk for poor recovery from COVID‐19.

**Table 1:** Demographic characteristics and COVID‐19 risk status of the UK sample

Age groups (N)		
18‐24:	N:13	
25‐34:	N: 79	
35‐44:	N: 175	
45‐54:	N: 205	
55‐64:	N: 124	
65‐74:	N: 38	
75‐84:	N: 11	
Gender (N)		
Female	N: 553	
Male	N: 88	
Other/Neither/Prefer not to say	N: 3	
Ethnicity (N)		
Asian	N: 11	
Black	N: 6	
Mixed	N: 12	
White	N: 612	
I prefer not to say	N: 3	
Disability (N)		
I self‐ identify as having a disability		
Yes	N: 73	
No	N: 568	
COVID‐19 symptoms		
Currently experiencing (N)	Yes: 10	No: 545
In the past (N)	Yes: 90	No: 635
Tested (N)	Yes: 9	No: 636
COVID‐19 risk status		
I have an underlying condition that increases my risk for COVID‐19 (N)		
No	N: 451	
Yes	N:188	
	Lung condition N: 88	
	BMI > 40: N = 34	
	Diabetes N: 22	
	A weakened immune system N: 20	
	Heart disease N: 8	
	Chronic kidney disease N: 4	
	Liver disease N: 1	
	Conditions affecting brain/ nerves N: 9	
	Problems with my spleen N: 2	

The vast majority of the sample had not been tested for COVID‐19 (N = 636) nor were they experiencing COVID‐19 symptoms at the time of completing the survey (N = 635). A small subgroup reported having experienced classic COVID‐19 symptoms (ie persistent cough and high temperature) in the past, but that they had not been hospitalized. The majority of participants considered themselves as not being in a higher risk group for COVID‐19 (N = 451). Of those who did report conditions that would place them at a higher risk for experiencing potentially serious COVID‐19 complications (N = 130), the majority reported a lung condition (N = 88) followed by obesity, as defined by a BMI > 40 (N = 34), and diabetes (N = 22).

### 3.3 Dimensions of comparative optimism

The results of the PCA revealed three clear factors explaining a total of 71.39% of the variance. The underlying rotated factors and the items’ respective factor loadings appear in Table 2.

**Table 2:** Principal component analysis results of the Comparative Optimism scale

Rotated component matrix			
	Factor 1 Hospitalization and recovery from COVID‐19	Factor 2 Current/imminent infection behaviours of self and other	Factor 3 Future infection and symptom development
Total variance explained (71.39%)	33.52%	24.83%	14.80%
Scale items			
Please think of the average person of your age and gender. Compared to them, how likely is it that…			
If you get infected (or re‐infected) with COVID‐19 you will need hospitalization?	0.862		
If you get infected (or re‐infected) with COVID‐19 you will need to be in an Intensive Care Unit (ICU)?	0.930		
If you get infected (or re‐infected) with COVID‐19 you will need to be in an Intensive Care Unit (ICU) and require a ventilator/intubation?	0.924		
If you get infected (or re‐infected) with COVID‐19 you will make a full recovery?	0.752		
You have in the last month accidentally infected others with COVID‐19?		0.735	
You will, within the next month, get infected (or re‐infected) with COVID‐19?		0.819	
You will, within the next month, accidentally infect others with COVID‐19?		0.853	
If you get infected (or re‐infected) with COVID‐19 you will develop symptoms?			0.724
You will, within the next year, get infected (or re‐infected) with COVID‐19?			0.698
You will, within the next year, infect others with COVID‐19?			0.546

**Figure 1:**
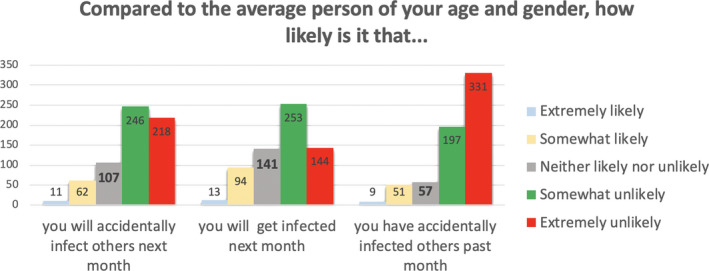
Frequencies of responses for items assessing risk of COVID‐19 infection (Subscale 1: Controllable COVID‐19 risks.)

**Figure 2:**
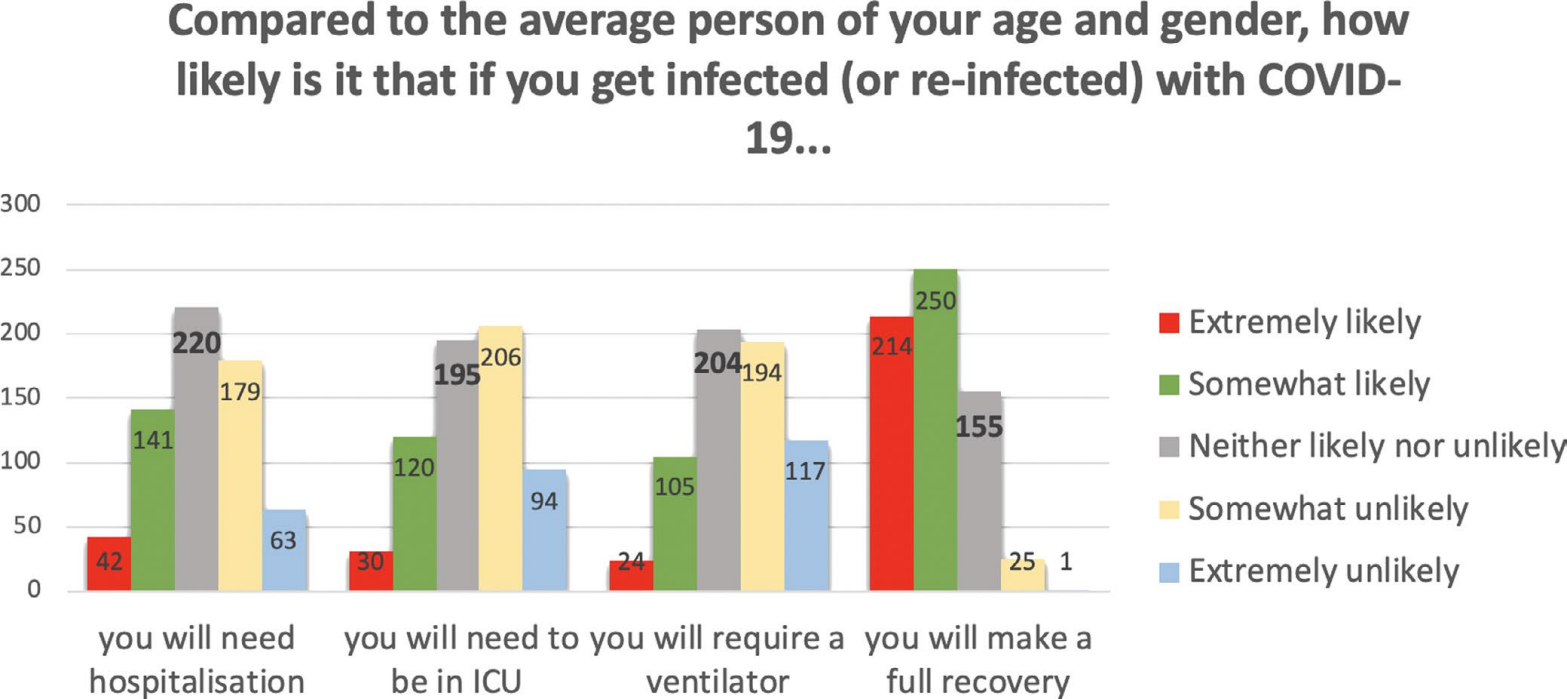
Frequencies of responses for items assessing risk of post‐COVID‐19 consequences (Subscale 2: Uncontrollable COVID‐19 risks)

**Figure 3:**
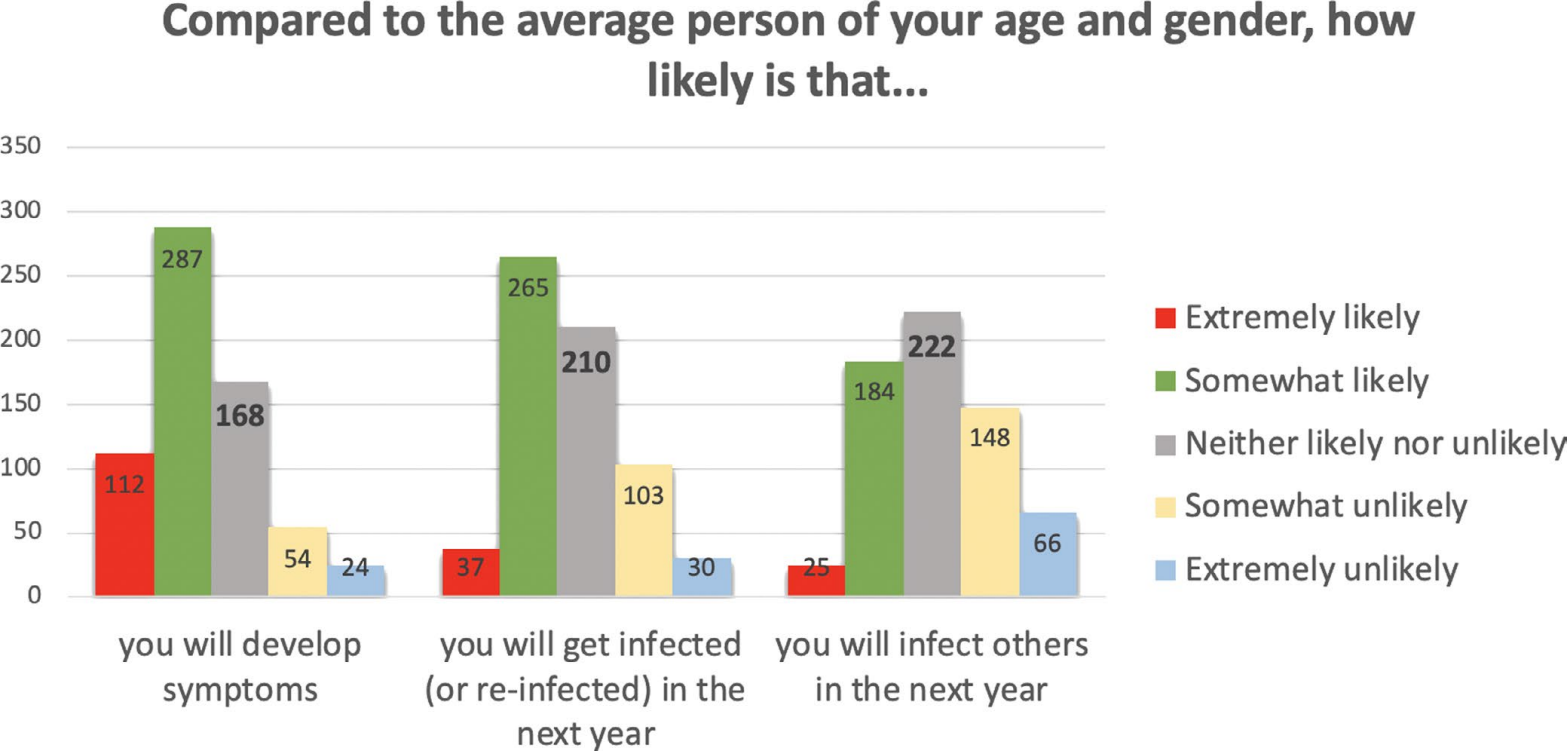
Frequencies of responses for items assessing future risks of COVID‐19 infection (Subscale 3: Future COVID‐19 risks)

We interpreted these factors as follows. Factor 1 relates to aspects of COVID‐19 that are outside the person’s immediate control, as they all relate to what may happen if and when one gets infected. Factor 2 relates to aspects of COVID‐19 that people arguably perceive as being controllable, that is, getting infected or infecting others in the past and the near future. Factor 3 mainly relates to aspects of COVID‐19 that may take place in the somewhat more distant future. The frequencies of responses within each of these three subscales, by item, appear in Figures 1, 2, 3.

### 3.4 Comparative optimism per Subscale

As shown in Figure 1, one‐third to almost half of the participants felt that as compared to the average other person of their age and gender, they were somewhat or extremely *unlikely* to need hospitalization, to find themselves in ICU, or to need a ventilator if they were infected by the virus, whereas considerably fewer felt that they were somewhat or extremely likely to experience those events. In addition, more than half believed they were somewhat or extremely *likely* to make a full recovery as compared to the average other person of their age and gender.

Figure 2 shows the vast majority of participants reporting they were somewhat or extremely *unlikely* to have accidentally infected others last month, to accidentally infect others next month or to get infected themselves next month, as compared to the average other person of their age and gender.

In sharp contrast, and as shown in Figure 3, about half of the participants thought that they were somewhat to extremely *likely* to get infected in the next year and, when infected, to develop COVID‐related symptoms as compared to the average other person of their age and gender.

### 3.5 Extent of comparative optimism

Having converted scores on each item into a continuous scale from −2 to + 2, we tested whether these risk perception scores were statistically different from a hypothesized population mean of zero. Negative scores indicated that the participant's risk estimate was comparatively pessimistic, positive scores suggested that the estimate was comparatively optimistic, whilst scores not significantly different from 0 suggested that the reported risk perceptions were no different from those of the average other person of the same age and gender. The results appear in Table 3.

**Table 3:** Means (*M*), standard deviations (SD) and significance (*P* values from single‐sample *t* tests analyses) of comparative optimism overall for each subscale and per item. Positive scores suggest comparative optimism, negative scores suggest comparative pessimism

Scale items Please think of the average person of your age and gender. Compared to them, how likely is it that…			
	Mean (*M*)	SD	Sig.
Overall Subscale 1 (hospitalization/recovery)	0.47	0.90	<.001
If you get infected (or re‐infected) with COVID‐19 you will need hospitalization?	0.12	1.06	<.003
If you get infected (or re‐infected) with COVID‐19 you will need to be in an Intensive Care Unit (ICU)?	0.33	1.08	<.001
If you get infected (or re‐infected) with COVID‐19 you will need to be in an Intensive Care Unit (ICU) and require a ventilator/ intubation?	0.43	1.08	<.001
If you get infected (or re‐infected) with COVID‐19 you will make a full recovery?	1.00	0.86	<.001
Overall Subscale 2 (past or imminent infection of self/ others)	0.93	0.85	<.001
You have in the last month accidentally infected others with COVID‐19?	1.22	0.98	<.001
You will, within the next month, get infected (or re‐infected) with COVID‐19?	0.65	1.04	<.001
You will, within the next month, accidentally infect others with COVID‐19?	0.93	1.02	<.001
Overall Subscale 3 (distant future infection of self/ others and symptoms development)	−0.28	0.73	<.001
If you get infected (or re‐infected) with COVID‐19 you will develop symptoms?	−0.63	0.99	<.001
You will, within the next year, get infected (or re‐infected) with COVID‐19?	−0.27	0.96	<.001
You will, within the next year, infect others with COVID‐19?	0.07	1.04	.08 NS

Table 3 shows significant comparative optimism for Subscale 1 and Subscale 2, as well as for each individual item of these subscales. Participants thought they were *unlikely* compared to the average other person to be hospitalized, to be admitted to ICU, to need a ventilator, to infect others and to get infected by others, either recently or in the next month. We found comparative pessimism for Subscale 3 and for two of its three individual items. Participants generally reported being more *likely* to get infected and develop symptoms in the next year compared to the average other person but felt they were no more likely than anyone else to infect others.

We compared the subscales that yielded comparative optimism through a repeated measures ANOVA with subscale (subscale 1 measuring uncontrollable aspects vs subscale 2 measuring controllable aspects) as a within‐subjects variable. Comparative optimism was significantly higher for subscale 2 than for subscale 1 (*F*_(1,642)_ = 81.37, *P* < .001), revealing that participants showed stronger comparative optimism for those aspects of COVID‐19 that may be deemed as controllable than for those considered controllable.

### 3.6 Gender effects

Because women were so greatly over‐represented in our sample, we wished to establish the generality of our findings across genders. There were no differences between men and women on Subscale 1 (M men = 0.51, M women = 0.47, *t*_(638)_ = 0.41, *P* = .683) and Subscale 2 (M men = 1.06, M women = 0.91, *t*_(638)_ = 1.58, *P* = .115). On Subscale 3, men were less pessimistic than women (M men = −0.08, M women = −0.31, *t*_(639)_ = 1.58, *P* < .001). The over‐representation of women in our sample thus does not detract from the validity of our findings.

### 3.7 Risk factor effects

We repeated all the analyses above excluding those participants who reported underlying conditions that might predispose them to worse outcomes if infected with COVID‐19. These analyses replicated the above findings. As a result, we have included the data from people reporting to be at higher risk for COVID‐19 complications.

## 4 DISCUSSION

On the basis of these data, we suggest that UK adults who meet the demographic characteristics of our sample display comparative optimism concerning many aspects of COVID‐19. Where participants showed comparative optimism its pattern was consistent with earlier findings showing that comparative optimism is stronger for controllable than for uncontrollable events.^6^, ^7^ Our participants overwhelmingly believed that as compared to people of their age and gender, they were somewhat or extremely unlikely to have accidentally infected people with COVID‐19 in the past and to infect others or get infected themselves in the next month. They were also comparatively optimistic, but to a lesser extent, about their likelihood of getting hospitalized due to COVID‐19, finding themselves in an ICU, being ventilated, and making a full recovery.

In contrast, participants showed comparative pessimism about COVID‐19 infections in the more distant future. As compared to the average person of their age and gender they felt *likely* to get infected by COVID‐19 in the next year and to develop COVID‐19‐related symptoms. This pattern is inconsistent with earlier findings showing greater comparative optimism for events that are further in the future than for nearer events.^21^, ^22^ However, such a finding supports earlier research that shows that people who have experienced some ill health tend to unduly exaggerate their future risk of experiencing further ill health.^23^ One important difference between COVID‐19 and other risks is that controlling the pandemic was very much placed in the hands of individuals restricting their lives in the UK—as seen in the slogan urging people to ‘Stay at home’. It is reasonable that participants would reason that in the long term, staying at home would be less possible, plausible or practical.^11^ Feeling that compliance with social distancing rules cannot be maintained indefinitely may thus explain these perceptions, in line with research showing that high prevalence negative events may engender comparative pessimism.^24^

We have thus established the presence of comparative optimism in relation to both controllable and uncontrollable aspects of COVID‐19. We have also found comparative pessimism concerning future infection and symptom development. Both comparative optimism and comparative pessimism may have important consequences for people’s psychological well‐being and their likelihood of engaging in risk behaviours or responding to further lockdown measures.

If people believe COVID‐19 ‘will not happen to me any time now’ or that they are unlikely to have infected others in the past or to do so in future, they may be more relaxed about lockdown advice. In an effort to make people look beyond their own risk (which for some age and gender groups may be lower than for other groups), most governments, including the UK government, have focused their communication about social distancing rules on how much these protect against infecting others. Unfortunately, having infected others and infecting others in the future are precisely the aspects of COVID‐19 on which we found the strongest comparative optimism—people think it is *unlikely* these will happen to them.

Equally, for people reporting comparative optimism for present and past COVID‐19 infection, these beliefs could fuel resistance to give up on lockdown—because to do so will place them amongst the very same ‘average others’ who—like them—have been unsuccessful in controlling the pandemic. Given that we have now established comparative optimism in relation to COVID‐19, future work should systematically explore how this thinking may influence behavioural outcomes such as returning to school, work and normal life.

There are limitations of this study which, although do not detract from the generalizability of the findings, should be noted. Firstly, the sample was predominantly White. Although this pattern is typical of wider online survey taking behaviour,^20^ it may well not represent the views of other ethnic groups. Our sample was also predominantly female, although that may be less of a limitation; our findings showed no gender differences in two subscales, entirely in line with previously reported work.^3^ The sole difference we observed involved men showing less comparative *pessimism* and thus being relatively more optimistic than women concerning their long term risk. If anything, then, our study may have underestimated comparative optimism by sampling fewer men. A further limitation of our study is that our participants have self‐selected to participate and that we have no means of estimating the participation rate. This is a methodological issue in all surveys conducted on‐line that use a sampling approach similar to ours. We are therefore confident that our results are no less robust and valid than other appropriately powered surveys in the field; the pattern of comparative optimism and pessimism that we have found is very much in line with patterns reported in previous work in the field of comparative optimism, and which used a range of recruitment strategies, response rates and methods of inquiry.^2^, ^3^

On the basis of the above, we conclude that UK adults may be comparatively optimistic about the chances of coming to harm due to COVID‐19 at the moment or having caused harm themselves previously. Future research is needed on the implications of comparatively optimistic thinking for future compliance with government guidelines on managing COVID‐19.

## CONFLICT OF INTEREST

The authors wish to declare no conflicts of interest.

## AUTHORS’ CONTRIBUTIONS

All authors have:

1. Made substantial contributions to conception and design and in the interpretation of data;
2. Been involved in drafting the manuscript or revising it critically for important intellectual content;
3. Given final approval of the version to be published. Each author should have participated sufficiently in the work to take public responsibility for appropriate portions of the content; and
4. Agreed to be accountable for all aspects of the work in ensuring that questions related to the accuracy or integrity of any part of the work are appropriately investigated and resolved.

## Data Availability

Data available on request from the authors.
